# A Hybrid Approach for MS Diagnosis Through Nonlinear EEG Descriptors and Metaheuristic Optimized Classification Learning

**DOI:** 10.1155/2022/5430528

**Published:** 2022-05-17

**Authors:** Elnaz Mohseni, Seyed Mahdi Moghaddasi

**Affiliations:** ^1^Department of Biomedical Engineering, Science and Research Branch, Islamic Azad University, Tehran, Iran; ^2^Biomedical Engineering Department, Semnan University, Semnan, Iran

## Abstract

Multiple sclerosis (MS), a disease of the central nervous system, affects the white matter of the brain. Neurologists interpret magnetic resonance images that are often complicated, time-consuming, and contradictory. Using EEG signals, this disease can be analyzed and diagnosed more accurately. However, it is imperative that MS be diagnosed by specialists using assistive technology. Until now, a few methods have been proposed in this field that are sometimes associated with different challenges in analysis. This paper presents a hybrid approach to MS diagnosis in order to decrease classification error rates. Using the proposed method, EEG descriptors in both the time and frequency domains are analyzed. After the feature extraction stage, a modified version of the ant colony optimization method (m-ACO) was used to select the appropriate subset of features. Then, the support vector machine is used to determine whether the disease exists. A metaheuristic algorithm adjusts the support vector machine's parameters to withstand overfitting challenges. Despite a limited number of input channels, significant classification accuracy has been achieved using wavelet analysis techniques, dividing all five subbands of EEG signals, signal windowing, and extracting efficient features from the data. Additionally, alpha, beta, and gamma bands of the signal are important, and the accuracy, sensitivity, and specificity levels are higher than 98.5%. Compared to similar MS diagnostic methods, the proposed method achieved significantly higher diagnostic accuracy. Application and implementation of this method can be effective in treating neurological diseases, including multiple sclerosis.

## 1. Introduction

As an autoimmune disorder that damages myelin sheaths in the brain and spinal cord, multiple sclerosis (MS) affects nerve cells in the brain and spinal cord [1, 2]. Damage to the nervous system's communication components can result in a wide variety of symptoms and physical difficulties [3, 4]. This condition most commonly affects people between the ages of 20 and 40. Based on disease distribution estimates across different countries, it is estimated that this disease is currently confined to Europe and the Far East [4]. Symptoms of MS are varied, and new symptoms occur recursively (i.e., multiple recurrences of the disease) or over time [5]. There may be complete disappearance of disease symptoms between relapses; however, permanent neurological problems persist, particularly as the disease advances [6]. Symptoms of the disease are usually recurrent and improve over time. Initially, seizures are almost completely recovered; neurological disabilities, however, may gradually persist with varying degrees of seizures [7]. Numerous studies have been conducted on this subject recently due to the importance of early diagnosis of MS. MRI is a noninvasive method for diagnosing and detecting mild cognitive disorders, Alzheimer's, Parkinson's, and other neurological diseases [8]. Lesions caused by diseases such as MS, Alzheimer's, and other prevalent brain diseases cannot always be distinguished from one another [9, 10]. Researchers are attempting to use MRI images to detect and diagnose neurological disorders with the least amount of error. Identifying the related lesions of this disease in its early stages will provide treatment options for the disease. Diagnoses and differentiations of brain lesions are currently done manually. To perform this procedure properly, a neurologist must spend a long time and be extremely precise [11]. A lack of contrast and resolution of images, as well as the similarity of the lesions created by the disease with other brain tissue, leads to differing interpretations of brain MRI images.

Electroencephalography (EEG) signals represent the brain state through body potentials. EEG-based computational methods are widely used to diagnose and identify various diseases [12]. Epilepsy diagnosis [13–15], seizures and strokes [16, 17], Alzheimer [18, 19], convulsions, depression [20], attention deficit disorder [21], biometric fields [22], and fatigue diagnosis [23, 24] are among the applications of EEG.

Numerous EEG-related analyses have used entropy calculation in recent years. By using this calculation, it is possible to identify the signal's complexity, instability, and nonlinearity [25]. The brain signals are considered to be functional electrophysiological effects of the brain [26–28], and recording the EEG signal can help differentiate pathologies, such as coma caused by hypoxia, convulsions, physiological recording, and similar cases [29]. There is a highly ambiguous correlation between brain signals and pathology in neuropsychological circumstances. Due to the complexity of the signals in people with MS, the ability to use EEG signals in MS diagnosis is less widely recognized.

In some studies of MS incidence, time limits and severity assessments have been made based on the study of the disease's incidence. When analyzing the nonstationary characteristics of an EEG signal, methods such as signal windowing can be effective. Simulated annealing (SA) and genetic algorithms enable detection of MS by analyzing EEG signals with better decision-making capabilities. A hybrid approach to MS diagnosis has been proposed, which analyzes both time and frequency domains of EEG. The ant colony optimization method (m-ACO) was used to select the appropriate subset of features after feature extraction. Using these features, the SVM determines the disease's presence. To avoid overfitting, a metaheuristic algorithm is used to adjust the SVM's parameters. One of the essential objectives of this work is to provide an automated method for diagnosing MS from EEG signals. The most important contributions of research are as follows:

- The possibility of analysis based on appropriate interpretation methods based on machine learning methods such as subband decomposition through frequency bands, windowing, linear and nonlinear feature extraction, feature selection, and classification will significantly reduce error disease diagnosis.
- Meanwhile, evolutionary algorithms can be very useful in improving analytical areas such as feature selection and classification.
- Our study uses subband signal decomposition, windowing, feature extraction based on different fractal dimension features, statistical and nonlinear features as in the study [30], feature selection using ant colony algorithm, and classifier parameters. We try to classify MS patients from healthy people, and in this way, we use signals with a minimum number of channels to record the signal.

The remainder of the paper is outlined as follows. In Section 2, related studies are discussed. The method in Section 3 provides an analysis of EEG signals for the diagnosis of MS. Findings from research are presented in Section 4. The results of the study will be discussed in Section 5, and the conclusions will be discussed in Section 6.

## 2. Related Work

Torabi et al. [30] proposed the classification scheme of two groups of healthy individuals and individuals with MS as a nonlinear model. Their study's main objective was to distinguish two groups of healthy volunteers and MS patients employing nonlinear features of EEG signals while conducting cognitive tasks. They applied nonlinear methods to extract the signal feature vector. They used criteria such as the *T*-test and the Bhattacharya test to decrease the dimension of the feature vector by scaling the features. In order to distinguish MS patients from healthy people, KNN and linear SVM methods were used. The maximum accuracy for diagnosing healthy people with MS before and after implementing the feature selection procedure has been evaluated to be 79.79% and 93.08%, respectively.

Kiiski et al. [31] have experimented Visual Evoked Potential (VEP) signals to assess the rate of disease progression in patients with MS. They studied 78 samples, of which 35 had MS, and 43 were healthy. The evoked potential signals varied from 0 to 700 milliseconds, and the study interval of the samples ranged from 1 to 13 months.

Arafat et al. [32] compared the brain signals of patients and healthy individuals with MS diseases. They used Virtual Reality- (VR-) based simulation in their investigations as rehabilitation systems. In this way, they performed dizziness, nausea, nausea, inattention, and the like for people and recorded brain signals. The connection between neurons is severed in MS disease, and thus, it is possible to analyze under experimental conditions as a stimulus procedure. They also recorded people's signals before stimulation and examined the relationship between the test and the process. However, they identified apparent differences between the characteristics of both groups and sought to generalize their diagnosing patients with MS.

Zipser et al. [33] also extracted information such as N45 or N100 amplitudes from EEG signals to diagnose MS patients. Their procedure helps differentiate between MS and healthy individuals and facilitates comparing the different amplitudes of the signals.

Similar studies have been performed to distinguish healthy individuals from patients with MS, which can be divided into phase-synchrony evaluation based on bivariate empirical mode decomposition (EMD) during a visual task [34], Computer-Aided Diagnosis (CAD) system based on phase to amplitude coupling in covert visual attention [35], and Optimization of Recurrence Quantification Analysis for Detecting the Presence of Multiple Sclerosis [36]. Each of them analyzed the MS and healthy people and implemented methods based on machine learning.

de Santiago et al. [37] proposed a model to identify individuals at different stages of MS progression using Multifocal VEP (mfVEP). They constructed feature vectors with characteristics about the latency intensity and singular values of the mfVEP signals. They also designed a hierarchical classifier (HC) and a flat multiclass classifier (FMC), and both were performed using the k-NN method.

In the study of Karaca et al. [38], bipolar channel coherence analysis of EEG signal obtained from MS patients and healthy individuals was performed. Therefore, feature extraction was conducted from specific frequency bands. In their work, the “Subspace Discriminant” classification was trained using the obtained features with 95.8% accuracy, and then the system was tested. As a result, accuracy, sensitivity, and specificity were achieved at 91.67%, 85.71%, and 100%, respectively.

Recent research has examined the application of new optimization algorithms to different learning methods and has been used in a variety of fields of medicine [39]. Using virtual reality technology and monitoring EEG signals before and after displaying diversified environments, Rezaee and Zolfaghari [40] developed a novel therapy approach for reducing stress levels in MS patients.

## 3. Proposed Method

**Figure 1:**
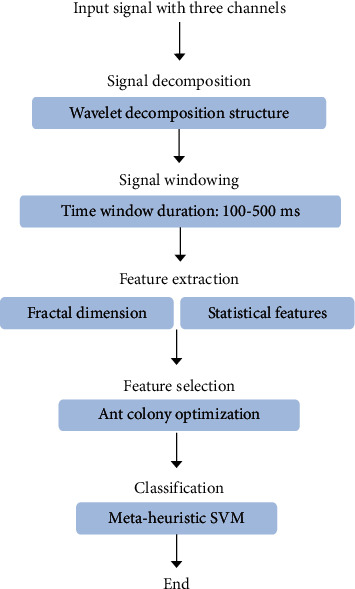
The overall schematic of proposed model.

**Figure 2:**
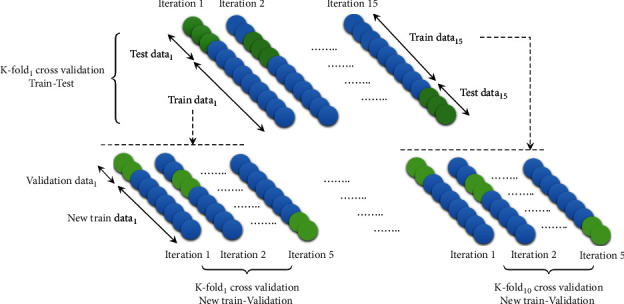
Data dividing based on *K*-fold cross validation.

**Figure 3:**
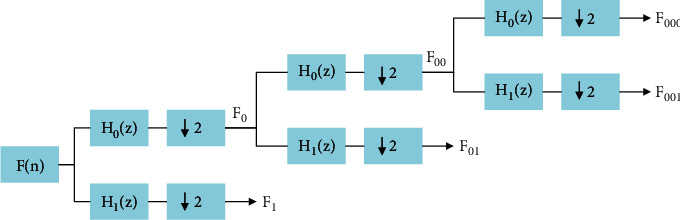
The decomposition procedure to separate subbands of EEG signals.

The proposed method consists of three steps of feature extraction, feature selection, and finally classification.  Figure 1 shows a schematic of the process steps. The data obtained is divided into three parts, that is, training, testing, and validation of data. The data obtained is divided into three parts, that is, training, testing and validation of data.  Figure 2 depicts how data is assigned in each step of the proposed algorithm.  Figure 3 illustrates the decomposition method for separating subbands of EEG signals.

### 3.1. Signal Windowing

By regarding the overlap between the frames, discontinued points on the signal are mitigated, which is a critical step in enhancing accuracy. Some studies do not employ windowing, preferring to utilize the whole signal to extract the feature, which impairs classification accuracy. The best potential output is estimated, based on which the frame length and the overlap between the frames will be prepared to obtain the best frame length. Arithmetic progression according to (1) is applied to achieve the number of frames based on time interval:

(1) $$\begin{array}{c} a_{n}=a_{1}+\left(n-1\right)d, \end{array}$$

where *a*_*n*_ is the last term of the signal on which the frame is placed (the last frame covering the signal), *a*_1_ is the first term of progression, *d* is the distance between the frames in the case of overlap, and finally *n* is the number of frames generated.

### 3.2. Feature Extraction

Power spectral density (PSD) estimation, known as one of the critical methods of EEG signal processing, has been used. In order to estimate autocorrelation sequences, nonparametric techniques such as Welch's method are used by calculating a Fast Fourier Transform (FFT). *F*_*yy*_^≈(*i*)^(*f*) the modified periodograms and *L* segments of the signal are taken into consideration when Welch's power spectrum is calculated [41]:

(2) $$\begin{array}{c} F_{yy}^{S}=\frac{1}{L}\sum_{i=0}^{L-1}\overset{\approx \left(i\right)}{P_{yy}\left(f\right)}. \end{array}$$

The sample entropy (SE) is the changed structure of the approximate entropy (AE). The AE estimates the complexity of a dynamic model. Low AE value regularly designates high low complexity and predictability of time series (TS). The SE for a signal with a length of *N* is provided by

(3) $$\begin{array}{c} \;SE\left(x,y\right)=\underset{N⟶\infty }{\lim}\left\{-\ln\frac{C^{x+1}\left(y\right)}{C^{x}\left(y\right)}\right\}. \end{array}$$

*C* ^*x*^(*y*) is the possibility of the relationship of two sequences in *m* points (i.e., based on distance), and also *C*^*x*+1^(*y*) is the possibility of two sequences in *x*+1 points. When *N* is a terminable capacity, the determined SE can be displayed as

(4) $$\begin{array}{c} \;SE\left(x,y\right)=-\ln\frac{C^{x+1}\left(y\right)}{C^{x}\left(y\right)}. \end{array}$$

In the above expression, *x* is the length of the investigated sequence, and *y* is the tolerance, on the basis of which a relationship between the sequences may occur and is usually chosen as 20% of *N*. Input signals are assumed to have a variance of 20% and 2 of the standard deviation. Spectral entropy (SpE) is another entropy described for the EEG signal, which is measured using the following formula:

(5) $$\begin{array}{c} \;H\left(f\right)=-{\left(\ln\left(N\right)\right)}^{-1}\sum_{i=1}^{N}p_{i}\ln\left(p_{i}\right), \end{array}$$

where *N* and *p*_*i*_ are the areas of the *i*_*th*_ frequency spectrum and the cumulative number of frequencies, and the aggregate of all *p*_*i*_ equals 1. The density of the power spectrum is achieved in each frequency period by determining the normalized value. The length of each period is equal to one spectral unit and is regularly considered to be 1 Hz. The high value of spectral entropy indicates the developed complexity of the frequency spectrum. Its low value designates the high density of the spectral power in a frequency period.

In fractals, deformation occurs frequently based on the starting position and is dependent on repetition. Katz and Higuchi models are the most prominent methods of generating random fractal dimensions.

Katz approach: in this system, *D* denotes the Euclidean distance within two samples:
  (6) $$\begin{array}{c} \;d=\underset{i,j}{\max}\left(D\left(P\left(i\right),P\left(j\right)\right)\right)\;i,\;j\in \left\{1,\ldots ,N\right\},\\ Len=\sum_{i=1}^{N-1}D\left(P\left(i\right),P\left(j\right)\right),\\ D\left(P\left(i\right),P\left(j\right)\right)=\sqrt{\left[{\left(x_{1}\left(i\right)-x_{2}\left(j\right)\right)}^{2}+{\left(y_{1}\left(i\right)-y_{2}\left(j\right)\right)}^{2}\right]}, \end{array}$$
where *d* denotes the max Euclidean distance of two continuous points or thicknesses. The *Len* additionally describes the entirety of all Euclidean distances for every two continuous points. Regarding the normalization factor *a* = *L*/*NL* and the parameter *d* as the fractal dimension, we will have
  (7) $$\begin{array}{c} \;D=\log\left(\frac{L}{a}\right){\left[\log\left(\frac{d}{a}\right)\right]}^{-1}=\log\left(N_{L}\right){\left[\log\left(N_{L}\right)+\log\left(\frac{d}{L}\right)\right]}^{-1}. \end{array}$$
Higuchi approach: the Higuchi approach presents an agreeable estimation of the fractal dimension for short sections of the signal with high velocity. A novel series of input series *x* is designed as equations (7) and (8) to determine the fractal dimension in this design:
  (8) $$\begin{array}{c} \;x=\;\left\{x\left(1\right),x\left(2\right),...,x\left(N\right)\right\}, \end{array}$$
  (9) $$\begin{array}{c} \;x_{m}^{k}=\left\{x\left(m\right),x\left(m+k\right),x\left(m+2k\right),\ldots ,x\left(m+\left\lfloor \frac{N-m}{k}\right\rfloor k\right)\right\}, \end{array}$$
where *m* represents the starting point of each series, and [.] is the integer of each number. The length of *L*_*m*_(*k*) for *x*_*m*_^*k*^ is equal to
  (10) $$\begin{array}{c} \;x_{m}^{k}=\left\{x\left(m\right),x\left(m+k\right),x\left(m+2k\right),\ldots ,x\left(m+\left\lfloor \frac{N-m}{k}\right\rfloor k\right)\right\}. \end{array}$$

### 3.3. Feature Selection

**Figure 4:**
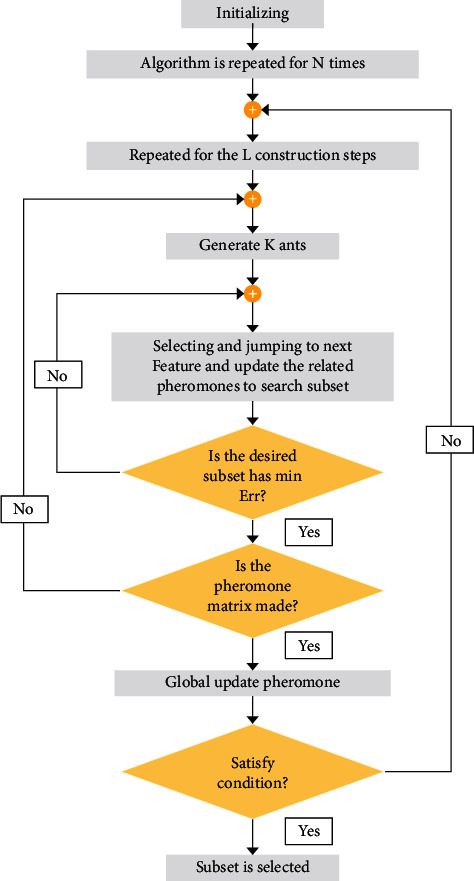
The feature selection procedure based on modified ACO (m-ACO).

The proposed method's feature selection has been carried out by getting inspiration from Rezaee and Zolfaghari method [40]. Their method was ant colony optimization (ACO), which was also considered in the present study to achieve the best subset of features, and the cost calculation in the fitness function was performed based on minimizing the classifier error. A constraint scheme called the subset size determination scheme is utilized in the m-ACO algorithm to address the problem of determining the subset sizes. As shown in Figure 4, we modified the procedure to select and jump to find the best global subset of features. The scheme controls the ants in guidable and reduced-size subsets.

Our modified algorithm procedure to obtain the optimal solution is as follows:

  Step-1: It has been assumed that *N* represents the feature set in the *D* data and *Cc* contains *d* separate classes (*c* = 1, 2,…, *d*). Moreover, *n* specifies the final number of features of *N*, and the pheromone trail of *τ* and exploratory information of *η* of all *n* features are assumed by assigning values equal to *τ* and *η*.
  Step-2: Measuring the information gain for *n* features using the gain measurement scheme.
  Step-3: Generating a *k*-member artificial set of ants assumed to be equal to *n*; *k*=*n*.
  Step-4: Making decisions regarding the initial size, “*r*” for each ant based on the problem size.
  Step-5: Completing the generation process to determine the status. The steps will continue if the subset generation is performed for all sets; otherwise, the procedure will return to step-4.
  Step-6: The subset of features is measured according to the subset evaluation scheme and the classifier performance measurement.
  Step-7: Select the best local subset and also the best global subset.
  Step-8: Evaluating the search termination conditions, including obtaining the optimal accuracy, the number of iterations threshold, or a specific execution time.
  Step-9: Updating *τ* and *η* values for all features.
  Step-10: Generating a new set of ants to reiterate the mentioned steps.

### 3.4. Optimized Support Vector Machine

The primary purpose of support vector machines (SVM) is to find an optimal hyperplane as a level of decision-making that maximizes the margin between the two classes. We map the data to another space by Φ kernel function to classify highly complex data. We represent the kernel function of the data from the input space to a space with higher dimensions, so that it is possible to separate the data in that space linearly. We have employed the Radial Basis Function (RBF) kernel trick for the SVM classifier. Typically, when a nonlinear kernel is selected, i.e., especially an RBF or polynomial kernel, the performance of the SVM in the conventional classification increases:

(11) $$\begin{array}{c} \;K\left(x,x_{i}\right)=\exp\left(-\frac{\gamma {\left\|x-x_{i}\right\|}^{2}}{2\sigma^{2}}\right). \end{array}$$

The best *γ* and *C* adjustments can be discovered by fixing one parameter and then performing a computationally demanding search within the range provided for the other parameter.

To select the best kernel RBF parameters, the grasshopper optimization algorithm (GOA) is used. Although GOA has proven to be effective in extracting valuable answers and ensuring accurate convergence, it is not a population-based strategy. As a result, the GOA possibility will lead to achieving the exact optimal point even in the increased dimensions of the functions. In this algorithm, the best answer always indicates the destination for the search grasshoppers. Thus, the grasshoppers do not deviate from the original optimal of the problem. In addition, the jumping behavior of the propellers in this algorithm allows it to search the exploration space around the optimal of the problem well and have good accuracy in obtaining the optimal. The steps of this algorithm are described according to Algorithm 1.

In the comfort zone update (an area with no attraction or repulsion), *C*_max_ represents the maximum value, *C*_min_ represents the minimum value, *l* represents the current (repetition) interactions, and *L* represents the maximum number of interactions. Furthermore, in updating the location of each locust, the first expression considers the position of the other locusts. It implements the interactions of the grasshoppers in nature, which are *ub*  _*d*_ upper limit and *lb*  _*d*_ lower limit in the *d* dimension space. The second expression ${\hat{T}}_{d}$ is the value of the *d*_th_ dimension in the target (i.e., the best answer ever seen) that mimics the grasshoppers' desire to move toward the food source. It should also be noted that the *c* parameter has been used twice for the following reasons: external *c* reduces the movement of the propellers around the target.

Moreover, *c* reduces the internal area of gravity, comfort zone, and repulsion area between the grasshoppers. The fit function is expressed according to

(12) $$\begin{array}{c} \text{ Fitness}=\left(1-\alpha \right)\times \left(\text{Accuracy}\right)+\alpha \times \left(1-G_{\text{mean}}\right), \end{array}$$

*α* indicates the obtained significance or error coefficient, and *G*_mean_ indicates the geometric mean when the classifier predicts the existence of MS classes (i.e., using RBF parameters). In order to expand the comfort zone to the new domain, equation (13) would look like this:

(13) $$\begin{array}{c} \;c=c_{\max}-l\left(\frac{c_{\max}-c_{\min}}{L}\right). \end{array}$$

This will update each grasshopper's position using the following:

(14) $$\begin{array}{c} \;x_{i}^{d}=c\left(\sum_{\begin{array}{c} j=1\\ j\pm i \end{array}}^{N}c\frac{Ub_{d}-lb_{d}}{2}s\left(\left|x_{j}^{d}-x_{i}^{d}\right|-\frac{x_{j}-x_{i}}{d_{ij}}\right)\right)+{\hat{T}}_{d}. \end{array}$$

## 4. Experimental Results

MATLAB R2019b was used to implement the proposed method in the Windows 10 operating system. Simulations were performed on a system with an Intel® CoreTM i5-8500 with 8 GB of RAM plus 16 GB of SSD RAM. Additional complementary software, such as SPSS, was also employed. Further, in the ACO algorithm, the classification error rate for all three simulation phases, including various signals of individuals, varied within the intervals of 0.056 to 0.062, 0.056 to 0.092, and 0.1 to 0.11. Initialization was required for the ACO algorithm. There are several significant initialization values, including the iteration number of the ACO algorithm, number of ants, constraint coefficients, control parameter of pheromone trail effect, control parameter of metaheuristic information effect, pheromone evaporation rate in the global update, the initial pheromone values, the number of decision variables, and the matrix size of the decision variables. In each step, the ACO algorithm selected the most optimized features and improved the detection algorithm by removing excess and repeated features. According to the mentioned points, the GOA parameters were acquired experimentally and through trial and error. The number of bits determined for displaying the setting parameters was 36.

To determine the appropriate initial population and to account for the large dimensions of the search space, an initially large population is generated as part of the optimization process. Then, the initial population is chosen based on the superior parameters in the RBF kernel. In the GOA, *α*, *C*_max,_*C*_min_, and max_iter_ parameters were set to be 1.25, 1, 0.00001, and 100, respectively.

In addition to the extracted features in the proposed method section, various statistical features in the time domain include features such as integral of the primary signal, absolute mean value, fundamental values of the third, fourth, and fifth-order temporal moments, root mean squares, orders of *V*, waveform length, and zero-crossing. Each of these features can be found in a different channel of EEG signals, which is a defect, and features vary for each channel. Furthermore, all the features extracted through the fractal dimension are around five features (the Katz dimension, the Higuchi dimension, the Petrosian dimension, the correlation dimension, and the self-similar fractal). On the basis of two feature models obtained from the two tables and also the acquired features from the proposed methods section, 31 features were generated. Decomposition of the signal revealed that alpha, beta, and gamma were the most effective bands. A total of 36 features were derived. The final selected vector produced an appropriate classification accuracy at a distance of 25 to 40% from the total features.

### 4.1. Dataset

The primary data is formed by a combination of a variety of MS-diagnosed individuals. Healthy individuals have also been examined in this study as manifest or control variables. The sampling process was performed using a probabilistic method. Furthermore, patients' characteristics were registered and collected by questionnaires. Moreover, the patients have undertaken a complete neurological examination. The obtained signals have been the outcome of performed records under different protocols in the laboratory. The EEG signal recording is performed such that the subject is resting with closed eyes. The electrodes are positioned on the scalp surface. A trained technician simultaneously applies an electrolyte gel to the skin according to International System 10–20. Two other electrodes (*A*1 and *A*2) are also connected to the earlobes, in addition to the main channels of *F*, *C*, *T*, *P*, and *O*. An electrode is located in the middle of the forehead (*Z*). In unipolar recording, this electrode serves as a reference electrode. The output EEG signals were collected through 4-minutes recordings (in case of removing artifacts such as blinking, average meaningful signals have a 1-minute duration), and the data are stored in “.txt” files. The patients' consent and approval of the attending physician have been acquired to conduct the study. The total number of study participants was 40; 19 subjects were clinically diagnosed with MS, and 21 were healthy. The mean age of the participants was 28.4 ± 5.2. The study sample included 23 women (14 MS-diagnosed and nine healthy individuals) and 17 men (5 MS-diagnosed and 12 healthy individuals). The signal sampling frequency was 250 Hz, and three channels were utilized to record the EEG signal.

Since some of the obtained signals of subjects were lengthy, synchronization of both types of received signals from the recording stages of participants was considered equal, which included the separated signals (240 seconds, in which 15 seconds of random signal recording has been separated from 60 seconds of meaningful signal recording). If the maximum length of each placed frame on each signal is considered to be 400 ms (selecting the size of this frame has been experimental and based on the mentioned methods in previous studies), around 50 frames will be acquired for a 15-second random signal selected from a 1-minute meaningful signal with 35% overlap rate among the windows. Five subbands and three channels have been investigated in each signal. In summation, a total of 500 meaningful segments will be obtained from each signal. According to the 250 Hz sampling frequency, the time step is equal to 0.004 s; therefore, for a 400 ms frame, 100000 samples will be generated that are appropriate for feature extracting of that segment. Considering the number of signal segments in both healthy and MS-diagnosed states, the *K*-fold cross-validation method (CV = 10) was utilized. Thus, the training samples have been equally sorted for EEG signals (56000 training signals and 4000 test signals).

### 4.2. Evaluations

The confusion matrices are presented using the 2 × 2 matrix, and the following various models are considered:

- *Model-1*: Temporal feature extraction and simple classification (SVM).
- *Model-2*: Frequency feature extraction and simple classification (SVM).
- *Model 3*: Temporal and frequency feature extraction and their aggregation and simple classification (SVM).
- *Model 4*: Temporal and frequency features extraction and their aggregation, simple feature selection, and classification (SVM).
- *Model 5*: Temporal and frequency feature extraction and their aggregation, optimized feature selection (m-ACO), and classification of SVM by GOA.

**Figure 5:**
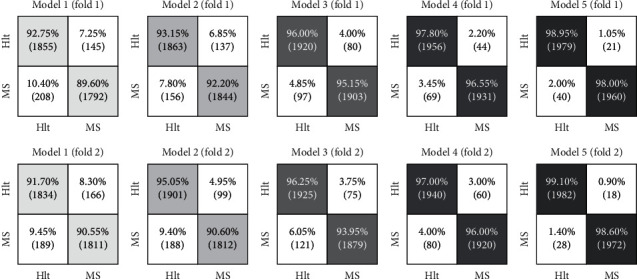
Two random folds of each model have been displayed. The improved procedure is shown in the performance of models with darker colors.

In all detection models, training and test data have been generated by the K-fold method (CV = 10), all subbands of the signal have been applied to the analysis, and the confusion matrix of the mean results of each tenfold for various models has been presented in Figure 5. The accuracy average is increased by 2 to 3% by adding the algorithms mentioned parts (gradually from model-1 to model-5).

**Figure 6:**
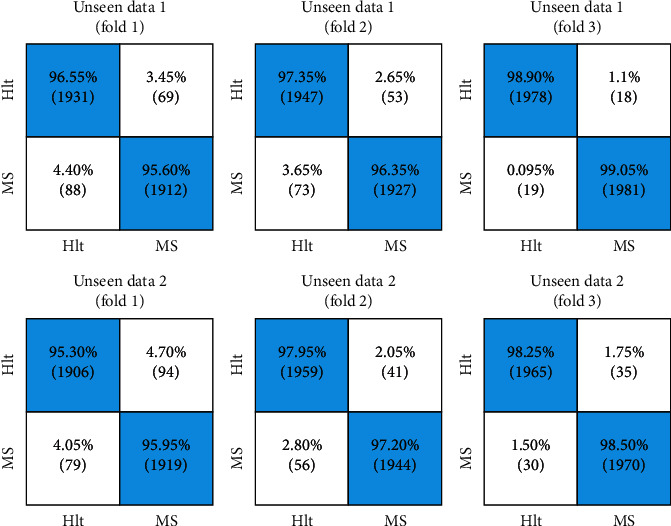
The proposed model in the diagnosis of MS disease is applied to two unseen data sets with 4000 signals (i.e., 2000 healthy and 2000 with MS disease). The results of applying the method are displayed in three random folds.

The proposed model of the research is model-5 utilized to diagnose the disease in each EEG signal by segmenting random signals. In this model, demonstrated in the last section of Figure 5, the highest accuracy level for a random fold is about 99.03%. Moreover, the sensitivity and specificity values have been reported by 98.90% and 99.18% values, respectively. In this model, the variation level is low, and the stability of solutions represents low solution dispersion. In Figure 6, the performance of the proposed model for MS diagnosis is shown that is based on three random folds of two groups of unseen EEG signals.

**Figure 7:**
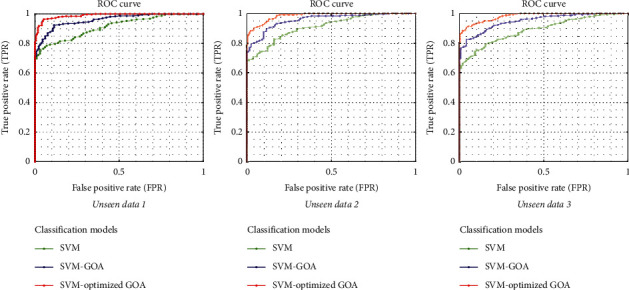
The shown ROC curves of the proposed method for MS diagnosis are based on various EEG signals. The performance of the proposed model is demonstrated by other similar methods such as SVM, SVM with GOA, and SVM with improved GOA.

In Figure 7, the receiver operating characteristic curve (ROC curve) of the proposed method for MS diagnosis has been shown that it is based on various EEG signals. In the figures shown, the performance of the proposed model is demonstrated by other similar methods such as SVM, SVM with GOA, and SVM with improved GOA. The performance of method SVM with optimized GOA is significantly improved compared to the other two techniques. As a result, the classification outcome shows that the area under the curve (AUC) is satisfactory. Hence, it inferred that the proposed approach could classify EEG signals related to healthy individuals and MS patients.

## 5. Discussion

In Table 1, the classification results of the *K*-fold method (*K* = 10) in 5 iterations and applying 3 feature levels (statistical, fractals, and hybrid) of classification mode have been demonstrated in comparison to the optimized SVM classification technique. As a result, the effective features based on the proposed feature selection method indicate a sufficient impact on the final classification accuracy and play an important role in the diagnosis of MS. Furthermore, this fact has been effective for the diagnosis of diseases, and 30% to 50% of the selected features have resulted in maximum classification accuracy.

**Figure 8:**
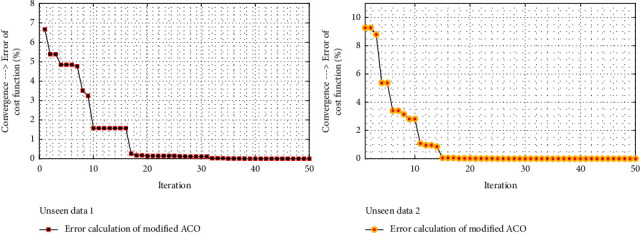
The considered convergence based on 50 limited iterations and the modified ACO for the two groups of unseen data is converged to the optimized value after 16 iterations.

In Figure 8, the convergence has been considered based on 50 limited iterations, and the modified ACO for two groups of unseen data is converged to the optimum value after 16 iterations.

If the feature volume reaches below 30% or higher than 60%, a particular reduction of accuracy will be observed. The reason for such decline is the existence of some excess or inappropriate features. This point has been presented in Figure 8. Although it seems that final accuracy and consequently accuracy resulting from features aggregation can be increased by extracting more features in the frequency domain, selecting 10 to 20 features results in favorable classification.

To compare the features effectiveness and the classification, the set of extracted features in the time domain and the frequency domain and for realizing a better understanding of the effective features on the correlation among brain signals with 5-iteration K-fold have been utilized to test the MS diagnosing accuracy. The accuracy of classification based on aggregated and selected features is higher than each presented model of time domain- and frequency domain-extracted features; moreover, the classification effect of the optimized support vector machine method has considerably improved the accuracy by 3% to 5%.

**Figure 9:**
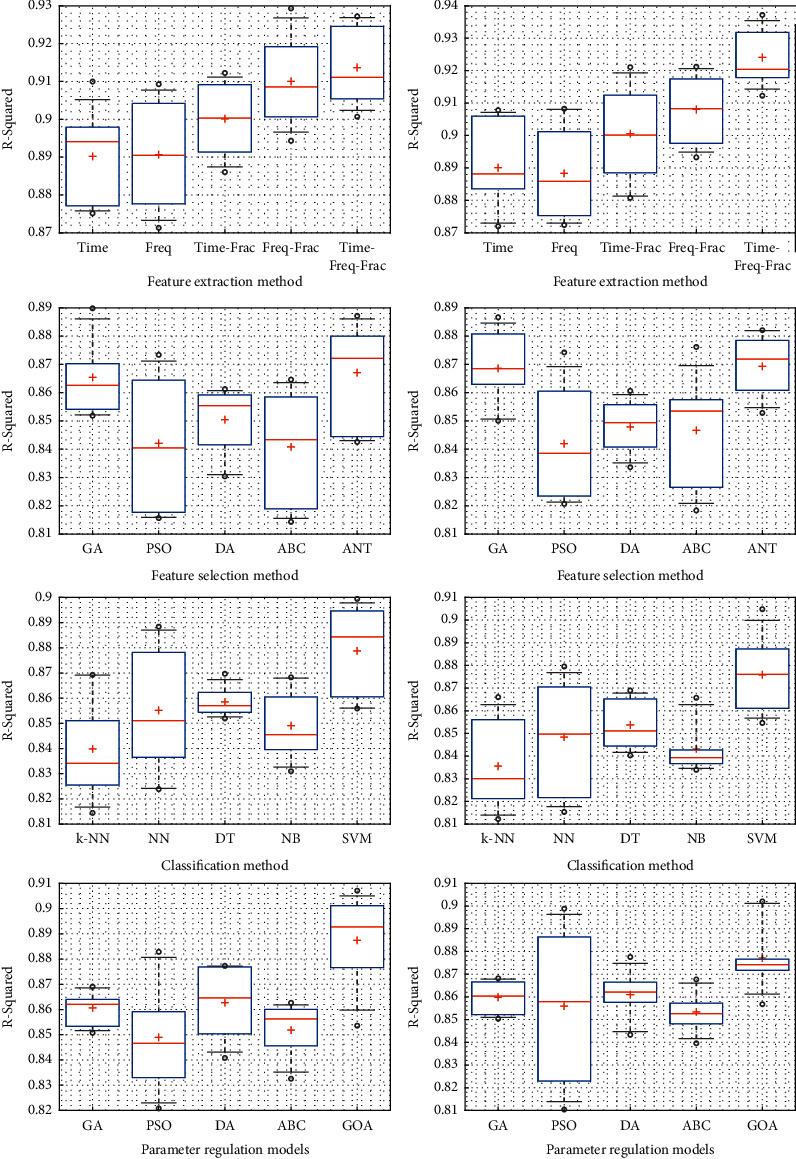
*R* ^2^ calculation to compare various types of feature extraction, feature selection, classification, and classification parameters optimization.

The *R*^2^ criterion is utilized to demonstrate the correlation amount. This criterion is the square of the existing relationship among the observed labels and estimated labels in the MS-diagnosis process. Equations (15) and (16) are used to acquire this criterion, which is efficient for investigating the extracted features.

(15) $$\begin{array}{c} \text{MSE}\left(y,\hat{y}\right)=\frac{1}{n}\sum_{i=1}^{n}{\left(y_{i}-\hat{y}\right)}^{2}, \end{array}$$

(16) $$\begin{array}{c} \;R^{2}\left(y,\hat{y}\right)=1-\frac{\sum_{i=1}^{n}{\left(y_{i}-\hat{y}\right)}^{2}}{\sum_{i=1}^{n}{\left(y_{i}-\bar{y}\right)}^{2}}, \end{array}$$

where *y*, $\hat{y}$, and $\bar{y}$ represent the main values, model-predicted values, and the mean values, respectively. Each of these values is considered according to the calculations resulting from the difference between the manually separated main model and the predicted values. In this regard, various tests have been performed. According to Figure 9, the box plots have been formed and examined to compare the approaches similar to the current approach in feature extraction, feature selection, classification, and classification optimization based on *R*^2^ calculation. Each test was repeated twice. The presented box plots in Figure 9 exhibit the extracted features examination, comparison of feature selection methods, comparison of SVM classification, and comparison of similar optimization methods in improving the vector machine hyperplane parameters. In Figure 9, the methods of time feature analysis, frequency feature analysis, time-fractal features analysis, frequency-fractal features analysis, and time-frequency-fractal features analysis have been compared, in which the dispersion of the last-mentioned model (time-frequency-fractal) utilized in this study is low. Moreover, the demonstrated *R*^2^ value of this method has been appropriately compared to its obtained value in other models.

Similar to the previous analysis in the second line with twice test repetition, the ACO in the feature selection method represents a higher relative mean *R*^2^ value in comparison to genetic algorithm, particle swarm optimization (PSO) method, differential evolution (DE) algorithm, and artificial bee colony (ABC) algorithm. Furthermore, its minimum value is higher than other methods and algorithms. However, its dispersion is higher than genetic and DE algorithms. Nevertheless, the ACO method has been selected since it has resulted in a higher mean *R*^2^ value.

The SVM classification method has been compared with similar techniques to make an accurate estimation of MS disease. In Figure 9, in the third line, the k-nearest neighbor classifier, feed-forward neural network (ff-NN), decision tree (DT), and Naive Bayes (NB) algorithms have been compared. The generated mean *R*^2^ value in the SVM method was higher than other methods. Although the dispersion value of NB and DT methods is less than the SVM method in the presented figures, it was inferred from different repetitions that the SVM kernel's dispersion level is lower than other techniques.

By performing other tests and comparing the accuracy-test results, we realize that the alpha, beta, and gamma bands have a significant influence on the signal. The result has been obtained directly from the repetition of the method's test using random 15- to 20-second segments of a meaningful 60-second signal. Previous approaches have examined the MS incidence; however, the length of the disease and the degree of severity of the disease have not been adequately considered. As well as detecting MS disease from EEG, the present study evaluated results by windowing methods. As a result of this point, the error rate in diagnosing MS disease significantly falls. The framing method may have been analyzed in previous methods for various lengths; however, based on current settings, it is clear that this method has significantly contributed to the process. Similar methods examine only the brain signals or exclusively the EEG signals. Analysis of these studies has not led to the identification of influential features that lead to correlation with responses.

Unlike the Torabi et al. study [30], which utilized the classification of two health and MS-diagnosed groups as a nonlinear model, this study is based on the extraction of linear frequency, temporal, and their aggregated features. Afterward, using the optimized SVM classification, which for the first time was optimized for EEG signals by the GOA, this study's method has resulted in higher accuracy than Torabi et al. study. In contrast to the proposed method of this study, studies [31, 32] and [42] have calculated high dispersion level outputs and solely have focused on monitoring the disease progress.

A real-time or near-real-time simulation of computational complexity using the proposed method was conducted in experiments. As opposed to providing structures for constructing features, our primary goal is to segment (windowing) and get the best classification result from EEG data. Increasing the number of signal components led to an increase in overlap and calculation time. It becomes difficult to analyze a nonstationary signal if it is not segmented. An optimal real-time system for neuromodulation feedback loops requires not only software-optimized methods, but also powerful hardware components the authors do not possess yet. In the offline training phase, our proposed model requires much time to learn. However, through the testing phase, the data processing was near real-time (i.e., the average processing time was 700 milliseconds). We will leverage IoT technology in future research initiatives. Since there is no need for additional hardware, the method can be applied in real time.

The method's performance varies to some extent as the level of noise increases (e.g., electrode impedance fluctuations, patient movement artifacts, etc.). Consequently, the robustness of the method is reduced when the amount of noise in the signal increases. Even though sufficient data is analyzed, it takes a considerable amount of training data to acquire the necessary features. Besides computational complexity, class imbalance, and uncertainty, there are other factors that affect the performance of proposed learning methods.

## 6. Conclusion

It takes expertise and time to make an accurate diagnosis of MS. A significant improvement in the accuracy of MS diagnosis is demonstrated here by analyzing EEG signals from health and MS-diagnosed individuals and utilizing methods such as linear and nonlinear signal describers, modified ant colony optimization, and an optimized SVM classification algorithm. The GOA has successfully improved the RBF kernel parameters for SVM. In addition, adding steps such as windowing for the nonstationary EEG signal have improved accuracy. In spite of the influence of the alpha, beta, and gamma sub-bands in signal analysis, the robustness of the technique with a random selection of 15 to 30-second segments of the EEG signal and the detecting capability of the algorithm have caused it to be appropriate for generalization. Furthermore, deep learning methods can improve the accuracy of the final MS diagnosis in this regard. The authors of this study will attempt to reduce the algorithm's computational complexity and execution time in the future.

## Figures and Tables

**Algorithm 1:**
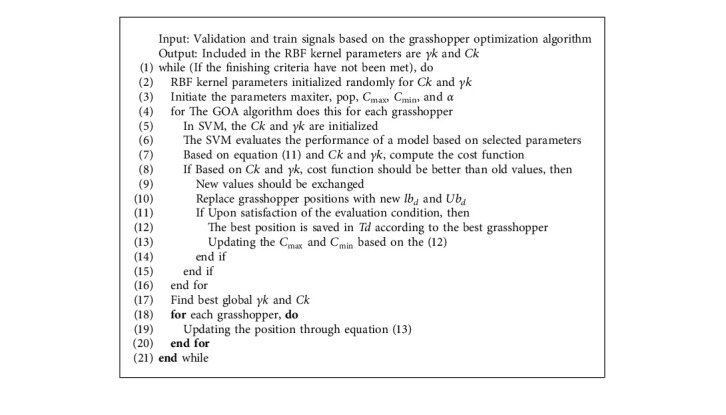
This algorithm shows the steps to find the parameters of the RBF kernel in SVM classifier using GOA.

**Table 1:** Assessments of accuracy in different data dividing situations and with/without feature selection strategy and change in the type of features. The bold values are the best measures achieved.

Data dividing	Type of features	Without feature selection			With feature selection		
		Best value	Mean value	Worst value	Best value	Mean value	Worst value
10-fold (1)	Statistical	0.93 ± (0.05)	0.90 ± (0.06)	0.88 ± (0.09)	0.96 ± (0.03)	0.94 ± (0.05)	0.93 ± (0.06)
	Fractals	0.91 ± (0.05)	0.90 ± (0.06)	0.88 ± (0.09)	0.97 ± (0.03)	0.94 ± (0.05)	0.93 ± (0.05)
	Hybrid	0.93 ± (0.04)	0.91 ± (0.05)	0.89 ± (0.08)	**0.98 ± (0.02)**	**0.95 ± (0.03)**	**0.94 ± (0.05)**

10-fold (2)	Statistical	0.93 ± (0.04)	0.92 ± (0.07)	0.88 ± (0.08)	0.96 ± (0.02)	0.95 ± (0.04)	0.94 ± (0.05)
	Fractals	0.93 ± (0.04)	0.91 ± (0.07)	0.88 ± (0.09)	0.98 ± (0.02)	0.95 ± (0.04)	0.94 ± (0.05)
	Hybrid	0.93 ± (0.05)	0.91 ± (0.06)	0.89 ± (0.08)	**0.98 ± (0.01)**	**0.96 ± (0.02)**	**0.94 ± (0.04)**

10-fold (3)	Statistical	0.92 ± (0.05)	0.90 ± (0.05)	0.87 ± (0.08)	0.95 ± (0.02)	0.94 ± (0.05)	0.93 ± (0.05)
	Fractals	0.92 ± (0.05)	0.91 ± (0.05)	0.88 ± (0.09)	0.97 ± (0.02)	0.95 ± (0.04)	**0.94 ± (0.04)**
	Hybrid	0.92 ± (0.05)	0.91 ± (0.04)	0.88 ± (0.08)	**0.98 ± (0.01)**	**0.97 ± (0.02)**	0.94 ± (0.05)

10-fold (4)	Statistical	0.93 ± (0.06)	0.91 ± (0.06)	0.88 ± (0.08)	0.96 ± (0.03)	0.95 ± (0.05)	**0.94 ± (0.03)**
	Fractals	0.92 ± (0.04)	0.90 ± (0.06)	0.89 ± (0.08)	0.97 ± (0.03)	0.95 ± (0.05)	0.93 ± (0.05)
	Hybrid	0.93 ± (0.05)	0.92 ± (0.06)	0.89 ± (0.07)	**0.98 ± (0.02)**	**0.97 ± (0.03)**	0.94 ± (0.04)

10-fold (5)	Statistical	0.92 ± (0.04)	0.91 ± (0.07)	0.87 ± (0.08)	0.96 ± (0.03)	0.94 ± (0.05)	0.93 ± (0.05)
	Fractals	0.93 ± (0.04)	0.90 ± (0.08)	0.88 ± (0.07)	0.97 ± (0.03)	0.96 ± (0.04)	0.94 ± (0.05)
	Hybrid	0.93 ± (0.03)	0.92 ± (0.07)	0.88 ± (0.07)	**0.98 ± (0.02)**	**0.97 ± (0.02)**	**0.95 ± (0.03)**

## Data Availability

All the data and codes are available through the corresponding author.
